# The theory of mind and human–robot trust repair

**DOI:** 10.1038/s41598-023-37032-0

**Published:** 2023-06-19

**Authors:** Connor Esterwood, Lionel P. Robert

**Affiliations:** 1 School of Information, University of Michigan, Ann Arbor, 48109 USA; 2 Robotics Department, University of Michigan, Ann Arbor, 48109 USA

**Keywords:** Mechanical engineering, Electrical and electronic engineering

## Abstract

Nothing is perfect and robots can make as many mistakes as any human, which can lead to a decrease in trust in them. However, it is possible, for robots to repair a human’s trust in them after they have made mistakes through various trust repair strategies such as apologies, denials, and promises. Presently, the efficacy of these trust repairs in the human–robot interaction literature has been mixed. One reason for this might be that humans have different perceptions of a robot’s mind. For example, some repairs may be more effective when humans believe that robots are capable of experiencing emotion. Likewise, other repairs might be more effective when humans believe robots possess intentionality. A key element that determines these beliefs is mind perception. Therefore understanding how mind perception impacts trust repair may be vital to understanding trust repair in human–robot interaction. To investigate this, we conducted a study involving 400 participants recruited via Amazon Mechanical Turk to determine whether mind perception influenced the effectiveness of three distinct repair strategies. The study employed an online platform where the robot and participant worked in a warehouse to pick and load 10 boxes. The robot made three mistakes over the course of the task and employed either a promise, denial, or apology after each mistake. Participants then rated their trust in the robot before and after it made the mistake. Results of this study indicated that overall, individual differences in mind perception are vital considerations when seeking to implement effective apologies and denials between humans and robots.

## Introduction

Humans and robots are increasingly expected to trust one another in order to accomplish tasks and achieve shared goals^1–3^. As a result, work arrangements between humans and robots has begun to resemble human work collaborations. In particular, humans are engaging in collaborative work settings with robots, which requires them to trust their robotic collaborators to effectively perform their job^4^. This is visible across an ever increasing range of domains from defense^5,6^, to logistics^7–9^, to retail^10,11^, and even to fast food^12^. In the case of logistics, warehouse robots search for and move goods while humans are tasked with verifying the goods retrieved in order to accomplish the shared goal of fulfilling orders^7,8^. This places robots in roles traditionally occupied by humans and largely leads to new collaborative work arrangements. Trust and maintaining it in a robot collaborator, which remains universally vital for work collaborations, is an especially important dynamic in these new work arrangements^13–17^.

Although trust is vital, it is not static but instead dynamic and changes based on whether or not the trustee has fulfilled their duties to the trustor. Trust can be defined as the “willingness of a party to be vulnerable to the actions of another party based on the expectation that the other will perform a particular action important to the trustor, irrespective of the ability to monitor or control that other party”^18,]^. Trust is dynamic in that it can increase when trustees are successful at performing tasks and meeting expectations. Alternatively, trust can decrease when trustees make mistakes or fail to meet their expectations^19^. This is true not only for humans but also for robots and AIs^19–23^. In the case of robots and AIs failures can occur for many reasons ranging from violating social norms to simply failing to retrieve a desired object^24^. Recent developments in the field, however, have increased the recoverability of robots across many different scenarios as it is now possible for robots and AIs to learn from their mistakes and adapt their behaviors to avoid future mistakes^25,26^. While this holds great promise, the initial decrease in trust caused by early errors often leads to disuse or an altogether rejection of robots and AIs as potential work collaborators^27^. This limits the possible benefits of deploying robots in work environments overall but also reduces the positive impacts that advances in learning and error recovery have on the efficacy of human–robot teams.

Fortunately, various trust repair strategies can be used to repair trust between humans and robots, namely, apologies, denials, and promises^22,28^. Apologies are expressions of remorse or regret^19,29,30^. For example, the phrase “I’m sorry” is an apology. Apologies largely rely on emotions and affect^19,29,31–33^ and are hypothesized to repair trust by changing the way that a trustor (individual bestowing trust) views a trustee (individual receiving trust)^30,34,35^. Apologies do so by acting as a form of social ritual that seeks to improve the social standing of the trustee, reestablish social expectations, and show respect to the trustor^36^. Denials are rejections of culpability coupled with one or more external reasons as to why a violation of trust was committed^20,]^. Denials are hypothesized to repair trust by changing the locus of causality associated with a trust violation^30,37^. In doing so, they shift blame for a negative event in order to clear a trustee of any wrongdoing thus bypassing the negative consequences of a trust violation. The third trust repair strategy, promises, are assertions by a trustee designed to convey positive intentions about future acts^38^. An example of a promise is the statement: “I promise I’ll do better next time.” Similar to apologies, promises are hypothesized to repair trust by changing the way that a trustor views a trustee, but, promises are distinct because they directly seek to change how the trustee is expected to act in the future^30,34–36,39,40^.

The effectiveness of each trust repair strategy in the human–robot interaction literature has been mixed^22^ and the degree of mind perception may help to explain why. Mind perception is the ascription of mental capacities by humans to other entities^41^. These entities can be non-humans such as animals, gadgets, and importantly robots^42–45^. Generally, mind perception can be considered a type of mentalizing^42,46^ and acts as a form of “pre-attributional process, identifying the kinds of causes that might explain or predict another’s behavior”^42,]^. In the context of trust repair, mind perception can influence the effectiveness of a trust repair strategy. Mind perception impacts the mental capacities that humans believe a particular agent possesses^42,43,47–52^. Therefore, it is possible that the degree of mind perceived can influence which trust repair strategies are seen as genuine or believable.

## Hypotheses

According to various works of literature, humans intuitively divide mind perception into at least two different categories: conscious experience and intentional agency^42,43,49–53^. Conscious experience—sometimes referred to as experiential mind^51^—encompasses the perception that an agent has the capacity for emotions. These can include emotions such as regret, sympathy, pride, or joy^54,55^. Conscious experience also encompasses the capacities for basic psychological states such as fear, hunger, thirst, and pain^41,42,49^. Intentional agency—sometimes referred to as agentic mind^51^—relates to the perception that an agent has the capacity to engage in goal-directed behavior, reasoned action, self-control, learning, and, strategic planning^42,49,50^.

The two dimensions of mind perceptions are not mutually exclusive and agents can be perceived as possessing various degrees of one with various degrees of the other^42,49,50^. In particular, robots have traditionally been ascribed to have lower levels of agency and experience when compared to humans^41,49–51^. Recent shifts in the designs of robots, however, have the potential to shift these ascriptions. In particular, humans can individually vary in the degree to which they see the same robot as possessing both intentional agency and conscious experience^51,56–58^. This has implications not only for how humans respond to robots overall but also for trust repair.

### Conscious experience and trust repair

A robot’s perceived capacity for conscious experience is likely to moderate the efficacy of various trust repair strategies as it sets the boundary for whether or not a particular repair strategy is seen as believable or valid from an emotional or affective standpoint. This is because, to a certain extent, all trust repairs rely on some degree of emotional appeals^19,28,29,31–33^. As such, this requires humans to believe that the robot is emotionally upset for violating the human’s trust. One main determinant of if a robot is capable of emotions relates directly to the perception of the robot’s ability to have conscious experiences^50,51,54,55^. More specifically, for a human to see a robot as capable of emotion, they must first ascribe that robot’s mind as possessing the capacity for conscious experience^50,51,54,55^. In doing so, this signals to the human the degree of sincerity attached to the robot’s message or in other words, to what degree the robot actually meant what it said. This is because, without the capacity for conscious experience, the robot will not be seen as being genuinely upset for violating the human’s trust. This will likely render any attempt at repairing trust appear ingenuine, making such attempts much less effective^31,32,59^. Therefore, for robots to be capable of effectively deploying trust repairs that rely on emotional or affective mechanisms—such as apologies—they must first be ascribed the capacity for conscious experience. This leads us to our first hypothesis:

#### **H1**

Trust repair strategies will be more effective when robots are seen as possessing higher rather than lower degree of conscious experience.

### Intentional agency and trust repair

A robot’s perceived capacity for intentional agency is likely to moderate the efficacy of different trust repair strategies as it sets the boundary for which of these repairs is seen as believable or valid from an intentionality standpoint. This is because to some extent all trust repair strategies rely on the perception that the agent seeking to restore trust has the intentionality to change their behavior^32,38^. One of the major determinants of if a robot is seen as possessing intentionality is the human’s perception of the robot’s mind. In particular, for a human to see a robot as capable of intentionality, they must first believe that robot’s mind as possessing the capacity for international agency^42,49–51^.

To be clear, the intention or agency of an agent speaks to the effort or motivation directed by an agent to change their behavior rather than the ability of an agent to actually perform better. An agent’s increase in effort can relate to but is distinct from the agent’s ability. In the case of human–robot trust repair, the effectiveness of any repair strategy relies on the degree to which a human believes that the robot has its own intentions. In cases where the human does not believe the robot has intentionality, trust repair strategies are likely to be interpreted as trite or meaningless automatic responses. This can ultimately lead to trust repair strategies being much less effective^38,60^. Therefore, for robots to be capable of deploying trust repairs that rely on intentions—such as promises—they must first be ascribed the capacity for intentional agency. This leads us to our second hypothesis:

#### **H2**

Trust repair strategies will be more effective when robots are seen as possessing a high rather than low degree of intentional agency.

## Methods

### Task and scenario

**Figure 1:**
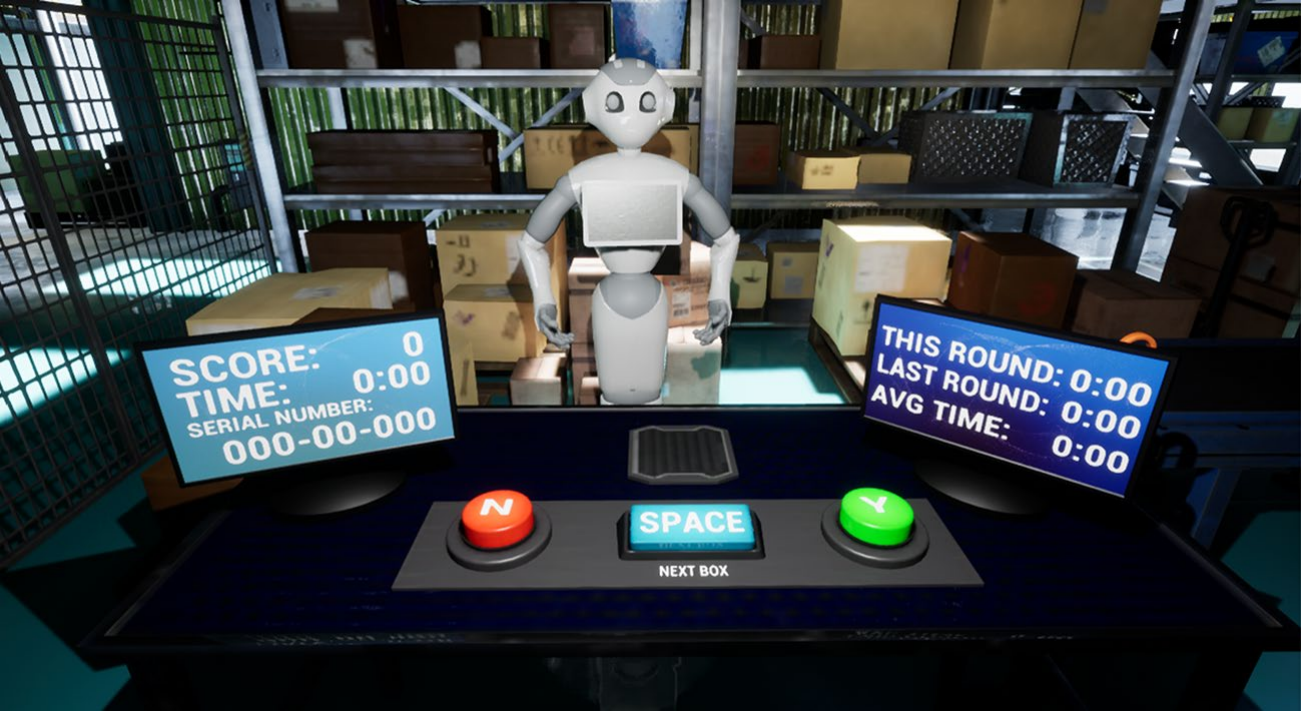
Environment and robot used from participants’ perspective.

To investigate the above hypotheses, this study used an open-source immersive virtual environment developed in the Unreal Engine 4.23 and deployed online^61^. Within this environment, participants were positioned behind a small table containing two computer monitors (see Fig. 1). From this position, participants engaged in 10 box-sorting and loading tasks where a human and robot worked as part of a team to process a series of boxes onto a nearby truck. Participants took on the role of “checker” and robots the role of “picker” for all 10 boxes. In these roles, the robot would pick a box from a nearby stack of boxes, present it to the human, and the human would determine whether this box was correct. Boxes were deemed correct if the serial number on the box matched the serial number displayed on a monitor. This monitor also displayed the amount of time taken to process these boxes as well as the participant’s score. In cases where the serial numbers matched, participants were instructed to approve the selected box, which triggered the robot to move the box to a nearby conveyor belt. In these instances, participants were granted 1 point. In cases where the serial numbers were different, participants were instructed to reject the selected box, which triggered the robot to place the box in a separate stack to the robot’s left. In such cases, participants were granted 1 point for catching the robot’s error. In cases where participants approved an incorrect box or rejected a correct box, they lost 1 point and the boxes were moved into the same stack as any previously rejected boxes.

Overall, 10 boxes were processed in the manner described, with the robot picking the wrong box at three evenly distributed trust violation events (box 3, box 6, and box 9). This produced a reliability rate of 70% based on previous work^62^. We included three errors based on the assumption that imperfect robots are likely to make mistakes more than once over the course of repeated interactions. The relatively short intervals between these mistakes was selected under a similar assumption that repeated failures would occur frequently when a robot is attempting something new and is engaged in learning or adaptation. Participants’ scores were used principally as a way to motivate them to engage in and complete the tasks and as a result a bonus payment of $5.00 was advertised and paid to participants who earned the most points during this study. In addition, these points also acted as a way of making trust violations more consequential which in turn makes trusting behaviors more salient. This was the case as trust violations could lead to points being deducted and no bonus payment being given. A visual illustration of the different ways in which boxes could be processed and their impact on the participant’s scores is visible in figure S1 in the supplemental materials associated with this paper.

This task and scenario was inspired by modern warehouse robots that pick goods based on orders and transport those goods (correct or incorrect) to humans for final packaging and quality assurance^7,63^. While we do not directly reproduce these interactions we do emulate the general flow of this work process and one possible place errors can occur within it. This was done to reduce the potential confounds that a direct reproduction of such interactions may have produced. Regardless, we feel that the results of this research in terms of empirical results could be applied in such environments.

### Experimental design

**Figure 2:**
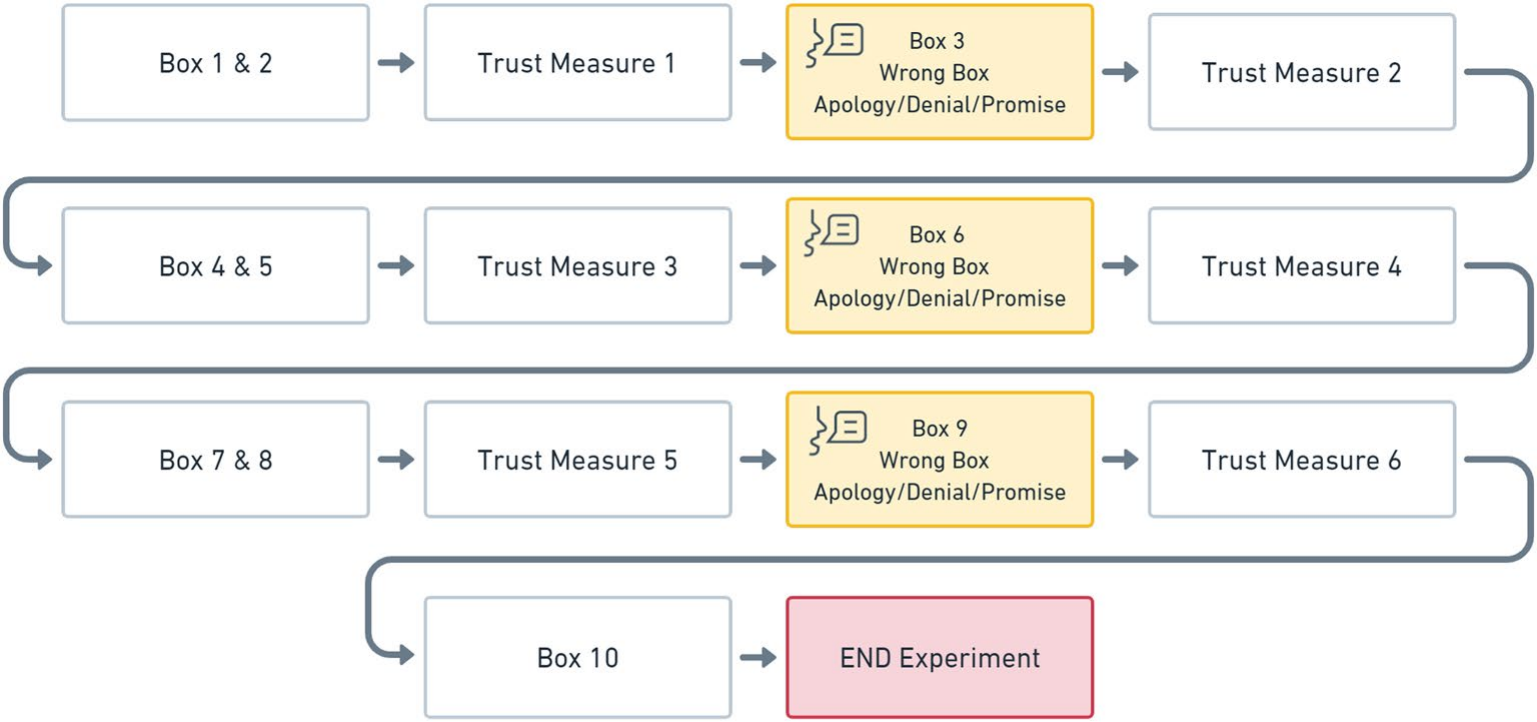
Flowchart illustrating study progression and timeline.

In this paper we designed and implemented a between-subjects study comprised of three experimental conditions and two control conditions. These conditions contained 80 subjects per cell. The experimental conditions differed by repair strategy where the robot deployed either apologies, promises, or denials after each time it provided an incorrect box to a participant (box 3, box 6, and box 9). This allowed us to measure the impact of these repairs over multiple violations as well as on average. In our two control conditions, the robot either performed perfectly at the task and always presented the correct box (no error condition) or remained silent during the study and deployed no trust repairs (no repair condition). These control conditions allowed us to measure the impact of the failures and use these data in the manipulation checks. A visual representation of our study’s design is presented in Fig. 2.

### Independent variables

The independent variables used in this study were the repair condition that participants were assigned to and human’s perceptions of the robot’s intentional agency and conscious experience. The different repair conditions used in this study were either apologies, denials, or promises. In the apology condition, the robot stated, “I’m sorry I got the wrong box that time.” In the denial condition, the robot stated, “I picked the correct box that time so something else must have gone wrong.” In the promise condition, the robot stated, “I’ll do better next time and get the right box.” Repair strategies varied by the assigned condition, and each participant was assigned to only one repair condition. These repairs were developed based on previous work^21,28^ and were designed to be the simplest form of an individual repair strategy rather than a combination of multiple strategies. In addition, these strategies were selected due to their wide application and popularity in the HRI and Human–Human literature^22^. Each strategy was deployed after the robot failed at processing the 3rd, 6th, and 9th box.

**Figure 3:**
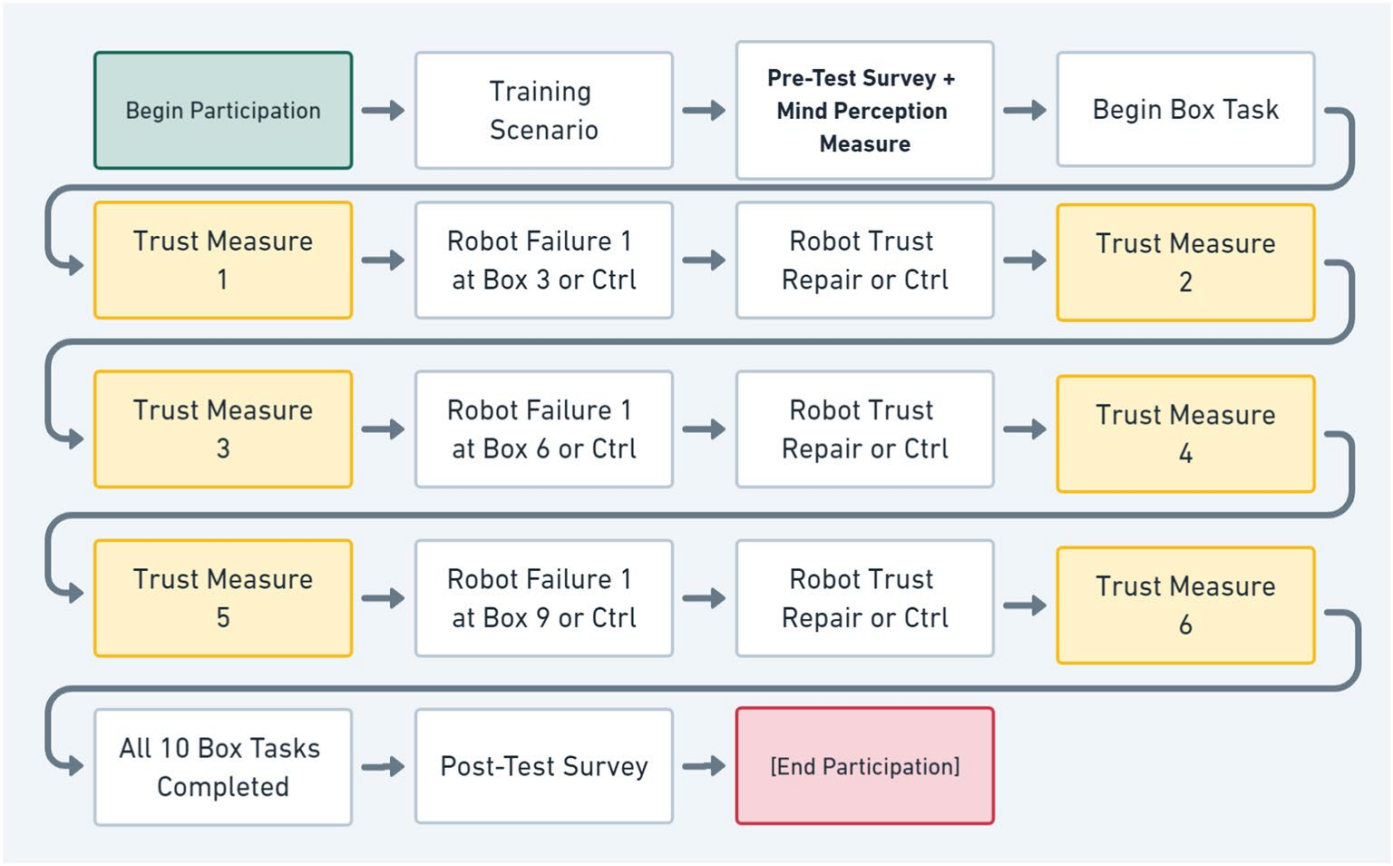
Flowchart illustrating measurement timeline.

Intentional agency and conscious experience were measured via a set of questionnaire items developed by Shanke et al.^51^. This measure was adopted for its relative simplicity, high overall reliability, and domain specific application to artificial agents. As a result, items were only minimally modified for this study and were found to be sufficiently reliable at $\alpha = 0.84$ for intentional agency and $\alpha = 0.97$ for conscious experience. This questionnaire consists of 6 items related to intentional agency and 11 items related to conscious experience. As visible in Fig. 3, we deployed this questionnaire after a brief training scenario that introduced participants to the environment, task, and robot they would be interacting with in the study. The specific items used in this measure are listed in the supplemental materials associated with this paper. By using this measure we were able to establish the degree to which each subject perceived the robot’s mind as possessing intentional agency and conscious experience.

### Co-variates and random effects

The co-variate used in this study was trust propensity. We included trust propensity in this fashion as it is likely to impact subjects’ pre-existing perspectives and will likely be linked to their willingness to trust in this study. We measured this as part of the pre-test survey and used an adapted 6-item instrument based on^64^. The specific items used in this measure are visible in the supplemental materials associated with this paper and were acceptable at $\alpha = 0.69$^65^. As a result, this measure was included in our analysis but not as a parameter/predictor and instead as a covariate (i.e. nuisance variable) that seeks to absorb elements of the variance inherent within the model.

Random effects are variables that “capture random or stochastic variability in the data that comes from different sources, such as participants”^66,]^. The random effect in this study was subject identification (ID). Subject IDs were assigned to participants randomly and each participant possessed a single unique ID. In linear mixed-effects models, subject ID represents a type of non-numerical blocking variable that defines which observations share a commonly realized random effect. This is possible as observations with the same subject ID come from the same subject allowing for a partial accounting of the variance across subjects. Importantly, subject ID in a linear mixed-effects model does not represent the outcome variable being simply regressed on the subject ID the way it does when employing generalized linear models. Instead, subject ID is simply used in the output to label the random effects due to the individual.

### Dependent variables

The dependent variable of interest in this study was participants’ trust change. We calculated this by subtracting the trust prior to a violation from the trust after the violation and repair. By examining trust change as opposed to trust at each of the six time points, we were able to establish the impact of a given repair strategy at a specific time. This then allows us to compare not only across time points where trust was expected to change but also between repairs and to determine if one repair strategy was more or less effective than another. To do this, we relied on a 3-item scale to measure trust adapted from Robert et al.^67^. In particular, we reworded three items from Robert et al.^67^ to better apply to the context of HRI. Two of these items stemmed from trusting intentions and one from trusting belief. These items collectively measured trust (not trustworthiness) and were found to possess sufficient reliability ($\alpha =0.84$) and have been validated in previous work^21^. We deployed this scale as a questionnaire at six points during the study, accompanied by attention-check questions. While other measures specific to robots exist (See:^68^), this measure was used instead due to the repeated measures aspect of this study and the desire to minimize interruption during tasks with longer questionnaires. Figure 3 illustrates when our selected trust measure was deployed while the items contained within the measure are listed in the supplemental materials associated with this paper.

### Participants

In total, we recruited 400 participants for this study. These participants were assigned to one of five conditions (four experimental and one control). Fifty-four percent (217) were male and the average age across participants was 36 years (standard deviation [SD] = 10.4 years). Participants were recruited via the Amazon Mechanical Turk platform and were required to be located in the United States of America and were compensated at a rate of $15/h, with the studies taking 15–25 min to complete. Amazon Mechanical Turk was used instead of in-person subjects due to various limitations at the time of the study and the impact of the COVID-19 pandemic. This research complied with the American Psychological Association Code of Ethics and was approved by the institutional review board at the University of Michigan, Ann Arbor (HUM00192093). Informed consent was gathered upon participants’ acceptance of the task on Amazon Mechanical Turk.

### Procedure

After participants were recruited, they were directed to participate in our training scenario. In this training scenario, participants were familiarized with the virtual environment. The training scenario demonstrated the box task used in this study by giving them one correct box and one incorrect box accompanied by tutorial dialogue boxes. The tutorial dialogue boxes communicated what button to press when the box was correct and what button to press when it was incorrect and explained the consequences of each action for the participant’s score. After this training scenario, participants were given the pre-test survey that gathered their basic demographic information and the degree to which they perceived the robot they interacted with during the training and would continue to interact with during the study as possessing intentional agency and conscious experience.

After they completed the training and pre-test questionnaire, participants were assigned one of our five conditions (no repair, no error, apology, denial, or promise) and progressed through the 10-box picking and checking task. After participants had completed processing all 10 boxes, they were asked to enter their worker ID for payment, which concluded their participation in the experiment. Throughout this process, we implemented quality and attention-check questions. These took the form of randomly placed questions requesting a specific response from participants. If participants provided incorrect responses to these questions, their participation was immediately ended, and their data were excluded from our analysis. This occurred for 296 subjects across our conditions in total. Subjects excluded from analysis were then replaced by new subjects until the desired sample size (80 per condition with 400 total) was met. The overall timeline of the study is summarized in Fig. 3.

### Data analysis

Data were exported directly from the survey platform used (Qualtrics), cleaned via Tableau Prep, and read into R via R studio. Analysis relied principally on the lme4, stats, jtools and emmeans packages in R^69–72^. The manipulation check was conducted via three pairwise t tests with a Bonferroni adjustment using the stats package. Specifically, we compared trust change after the first, second, and third violations in the no repair and no error conditions. Pairwise t tests are a specific type of pairwise comparison that calculates pairwise comparisons between group levels with corrections for multiple testing^69^. The specific adjustment used in this case was a Bonferroni adjustment which multiplies the p-values produced in the pairwise comparison by the number of comparisons in order to reduce the probability of identifying significant results that do not exist^69,73^. Given that this analysis involved conducting multiple pairwise t tests this correction was deemed appropriate.

For the main analysis, we constructed and compared the five mixed linear effects models. Mixed linear effects models are extensions of traditional linear models that allow for the examination of both between-subjects effects (i.e. fixed effect) and within-subjects effects (i.e. random effects)^66,70,73^. Furthermore, mixed linear effects models also permit the exploration of alternative covariance structures on which one can model data with between and within subjects effects^73,]^. We opted to use this statistical approach as it is capable of encompassing more than just fixed effects consistent with our design and goals of this study. We developed these models using lme4^70^ and compared them via a likelihood ratio test in emmeans to select the most appropriate model for this analysis. Likelihood ratio tests are “standard statistical test for comparing the goodness of fit of two nested models”^66,]^. These allow for the comparison of nested mixed linear effects models and the selection of the most appropriate of these models rather than risking “cherry picking” the best model for a specific set of hypotheses based on a range of different parameters. We opted to use this form of model comparison as each subsequent model—Baseline Model, Reduced Model 1, Reduced Model 2, etc.—included elements of the previous model (i.e. were nested).

After a comparison of these models, we examined the first reduced model’s two-way interaction between perceptions of a robot’s conscious experience and trust repair strategy via a simple slopes analysis and interaction plot. This model was selected as all subsequent models did not produce a significantly better fit for the data (See: Model Comparisons in Table 2). Simple slopes analyses are a method by which one can probe interaction effects in a linear regression^71,74^. In particular, one can construct confidence intervals for simple slope estimates that can indicate if slopes are significantly different from zero^75,76^. Often this includes the production of an interaction plot which displays one variable on the *x* axis, a dependent variable on the *y* axis and draws one or more line for the means of each level of one additional (often categorical) variable^77^. Both approaches relied on the use of jtools in R^71^. After this we also conducted a pairwise comparison of slopes via the emmeans package in R^72^. This allowed us to compare the slopes to each other and determine if significant differences emerged between slopes rather than only if those slopes were significantly different from zero^72^.

In sum, the approach outlined above allowed us to explore the interactions of interest in this paper that were produced by the mixed linear effects model that best suited the data. The data used in our analysis and associated code can be located at https://zenodo.org/record/8050885. Furthermore, the simulation and associated UE4 resources used are currently available for future researchers at no cost under a non-commercial license (see^61^). The following section presents the results of this analysis.

## Results

### Manipulation check

**Figure 4:**
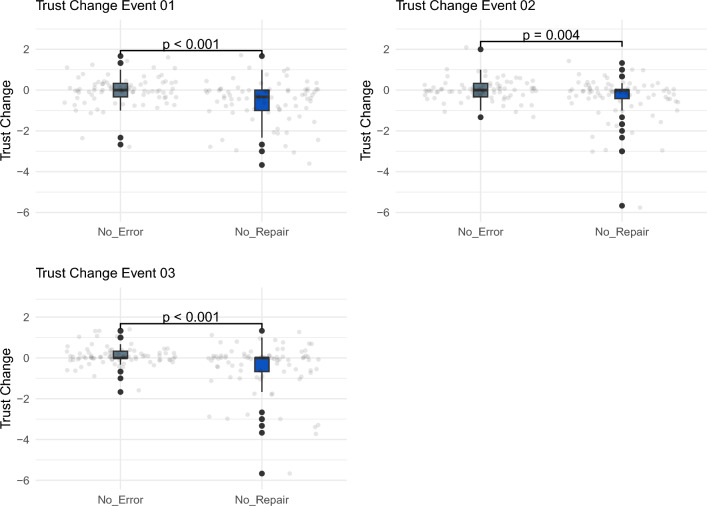
Box plots showing results of manipulation check across all three trust change events.

We conducted a manipulation check in this study with the goal of verifying that the trust violations used in this study did indeed violate trust. This was necessary as only when trust is effectively violated can the efficacy of a given trust repair be assessed. To do this, we compared trust change in a condition without violations (no error) to trust change in a condition with violations but no repairs (no repair). Results indicated that our manipulations were effective at all three trust change events, as shown in Fig. 4. This was the case as trust in the no error condition was significantly higher ($P<0.005$) than trust in the no repair condition across all three time points. From this we can conclude that the presence of trust violations decreased trust therefore allowing us to explore how this decrease can be mitigated via different repair strategies and the potential moderating effect of mind perception.

### Trust repairs and mind perception over multiple violations

After determining whether our manipulations were effective, we began the process of testing our hypotheses by constructing five mixed linear models, namely 1 baseline model, 3 reduced models, and 1 full model. Table 1 details the composition of these models and their results are fully detailed in the supplemental documentation associated with this publication. By constructing multiple models we facilitated the comparison of these models and selection of only the most appropriate model for our data and subsequent analysis. To accomplish this, we used likelihood ratio tests to compare nested mixed effects models to determine which of the possible models presented was a best fit for the data. This is often done to determine which specific parameters (i.e. predictors) one should include in their model and prevents over-fitting or under-fitting the data by including too many or too few of these parameters^78^. This allows us to select a model on the basis of fit rather than “cherry picking” a model most suited to our hypotheses or one that has the most statistically significant results.

**Table 1:** Table summarizing the composition of the models compared in this study.

Main effects	Interaction effects	Covariates	Random effects
*Baseline model*			
Repair strategy		Trust propensity	Subject ID
Intentional agency			
Conscious experience			
Violation event			
*Reduced model 01*			
Repair strategy	Repair strategy × Intentional agency	Trust propensity	Subject ID
Intentional agency	Repair strategy × Conscious experience		
Conscious experience			
Violation event			
*Reduced model 02*			
Repair strategy	Repair strategy × Intentional agency	Trust propensity	Subject ID
Intentional agency	Repair strategy × Conscious experience		
Conscious experience	Violation event × Intentional agency		
Violation event	Violation event × Conscious experience		
*Reduced model 03*			
Repair strategy	Repair strategy × Intentional agency	Trust propensity	Subject ID
Intentional agency	Repair strategy × Conscious experience		
Conscious experience	Violation event × Intentional agency		
Violation event	Violation event × Conscious experience		
	Repair strategy × Violation event		
*Full model*			
Repair strategy	Repair strategy × Intentional agency	Trust propensity	Subject ID
Intentional agency	Repair strategy × Conscious experience		
Conscious experience	Violation event × Intentional agency		
Violation event	Violation event × Conscious experience		
	Repair strategy × Violation event		
	Repair strategy × Intentional agency × Violation event		
	Repair strategy × Conscious experience × Violation event		

For the likelihood ratio test used in this study, we compared the nested baseline model to the first reduced model, the first reduced model to the second reduced model, and finally the third reduced model to the full model. We did this to determine whether the inclusion of the additional interaction terms significantly improved the model performance (i.e. fit). Results of these comparisons are presented in Table 2. These results indicated that the additional terms present in the first reduced model (reduced model 1) led to a better fit ($\chi ^2$) than the baseline model. However, subsequent models did not outperform the first reduced model. The model comparisons indicated that the first reduced model (reduced model 1) should be used for analysis and probing interactions. As a result, we used reduced model 1 for the remainder of our analyses. Within this model a significant effect for violation event was observed ($p=0.02$) where trust change at the second violation event was significantly different from change at the first violation event. Additionally, a significant two-way interaction effect between apologies and perceived conscious experience ($p= 0.03$) was also observed. These results are further examined in the subsequent sections of this paper and are fully detailed in Table 3.

**Table 2:** Results of likelihood ratio test comparing the models constructed for this study.

Model comparisons						
Model	npar	AIC	LL	$\chi ^2$	*df*	*p*
Baseline	11	2698.8	− 1338.4	NA	NA	NA
Reduced 1 vs. baseline	17	2697.9	− 1331.9	12.95	6	0.04
Reduced 1 vs. reduced 2	21	2701.3	− 1329.6	4.58	4	0.33
Reduced 2 vs. reduced 3	27	2706	− 1326	7.32	6	0.29
Reduced 3 vs. full model	39	2710	− 1316	19.74	12	0.07

**Table 3:** Results of reduced model 1 predicting trust change.

Reduced model 01			
Predictors	Estimates	CI	*p*
(Intercept)	0.48	− 0.73 to 1.69	0.432
Repair [denial]	− 0.59	− 2.16 to 0.98	0.463
Repair [apology]	0.05	− 1.32 to 1.42	0.944
Repair [promise]	0.1	− 1.41 to 1.62	0.895
Intentional agency	0.07	− 0.22 to 0.35	0.636
Conscious experience	0.15	− 0.03 to 0.32	0.108
Violation event [2]	0.14	0.02 to 0.26	**0.02**
Violation event [3]	0.07	− 0.04 to 0.19	0.21
Trust propensity	− 0.38	− 0.55 to − 0.22	<**0.001**
Repair [denial] $\times$ Intentional agency	− 0.08	− 0.48 to 0.31	0.675
Repair [apology] $\times$ Intentional agency	− 0.25	− 0.63 to 0.13	0.198
condition [promise] $\times$ Intentional agency	0.12	− 0.31 to 0.55	0.583
Repair [denial] $\times$ Conscious experience	0.17	− 0.06 to 0.41	0.147
Repair [apology] $\times$ Conscious experience	0.29	0.04 to 0.54	**0.025**
Repair [promise] $\times$ Conscious experience	− 0.16	− 0.45 to 0.13	0.276
*Random effects*			
$\sigma ^2$	0.57		
$\tau _{00}$ Sub ID	0.71		
ICC	0.55		
N Sub ID	320		
Observations	960		
Marginal/conditional R2	0.185/0.637		

Significant values are in [bold].

#### Conscious experience and apologies

**Figure 5:**
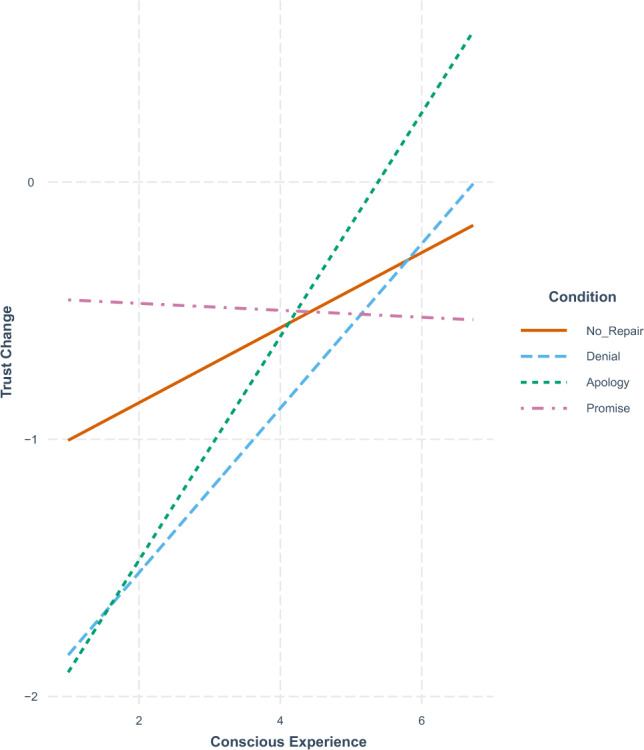
Visual representation of slopes for three-way interaction between **conscious experience**, repair strategy, and violation event.

Given the significant two-way interaction effect between apologies and perceptions of a robot’s conscious experience for reduced model 1 in Table 2, we conducted a series of additional statistical tests to probe this interaction. To do so we first conducted a simple slopes analysis. Simple slopes analysis allows us to determine whether any of the slopes within an interaction are significantly different from zero. Results of this analysis indicated that the slopes of the denial ($p < 0.001$), and apology ($p < 0.001$) conditions were significant. Next, we compared slopes across repair strategies. To do this we conducted a pairwise comparison of slopes for apologies, denials, and promises with a Tukey adjustment^72,79,80^. Results of this analysis showed a significant difference between the slopes of apologies and promises ($p = 0.01$) but no significant difference between the slopes of denials and promises ($p = 0.06$). Overall these results support our first hypothesis but only partially. Specifically, apologies and denials appear to be more effective when subjects ascribed the robot greater levels of conscious experience and less effective when subjects ascribed the robot lower levels of conscious experience. Figure 5 illustrates these interactions while Table 4 summarizes the results of the simple slopes analysis, and Table 5 summarizes the results of our pairwise comparison.

**Table 4:** Results of simple slope analysis examining slopes of the two-way interaction between perceptions of a robot’s conscious experience and repair condition.

Condition	Trend	SE	*df*	t.ratio	*p* value
No_Repair	0.15	0.09	307	1.61	0.11
Denial	0.32	0.08	307	4.00	< 0.001
Apology	0.44	0.09	307	4.68	< 0.001
Promise	− 0.01	0.12	307	− 0.12	0.91

**Table 5:** Results of a pairwise comparison of slopes for the two-way interaction between perceptions of a robot’s conscious experience and repair condition.

Contrast	Estimate	SE	*df*	t.ratio	*p* value
Denial–apology	− 0.12	0.13	230	− 0.91	0.64
Denial–promise	0.33	0.15	230	2.28	0.06
Apology–promise	0.45	0.16	230	2.90	0.01

## Discussion

Overall the results of this study provide valuable insights into the relationship between mind perception and trust repair in human–robot interaction. In particular, this study highlighted that perceptions of a robot’s conscious experience moderate the impact of apologies and denials. In doing so, this study helps to explain *when* apologies and denials are likely to be effective at repairing trust for robots providing a significant contribution to the existing literature. This, in combination with careful assessments of timing^81,82^ and violation type^83,84^, could lead to more effective HRI trust repair. Contrary, the study found no evidence that perceptions of a robot’s intentional agency influence the effectiveness of any trust repair strategy. Below we discuss the implications for existing theories related to trust repair and mind perception in robots.

First, this paper demonstrates the theoretical distinctions between mind perception as conscious experience and mind perception as intentional agency, and their unique impacts on human–robot trust repair. Specifically, conscious experience enhances the effectiveness of apologies and denials, while intentional agency does not. The increased effectiveness of apologies and denials due to conscious experience may be attributed to the perception that the robot is more sincere. Previous research has established a connection between mind perception and perceptions of sincerity^85^. Sincerity has also been shown to be a crucial factor in the efficacy of trust repair strategies^86–90^. For instance, apologies from individuals perceived as sincere have a more positive impact compared to those perceived as less sincere^38,86–89,91^. Similarly, denials from individuals perceived as sincere are also more likely to be believed and therefore effective^19,90^. To this end, this study reveals the theoretical importance of separating the dimensions of mind perception to understand their influence on HRI trust repair.

Conversely, in the case of intentional agency, we did not observe any significant interaction. One possible explanation for this could be found in prior research that has established a link between intentional agency, attribution of blame, and moral responsibility^43,92^. Specifically, if a robot is perceived as possessing higher levels of intentional agency, it may be viewed as more responsible for a trust violation. Moreover, increased perceptions of intentional agency could be counterproductive, as it may reduce perceptions of sincerity^92^. Therefore, in the context of HRI trust repair, intentional agency could be recognized as a potential obstacle to overcome, rather than an advantage to leverage. However, it is important to note that intentional agency may actually enhance trust when the robot fulfills its expectations, rather than violates them. This is because humans are also likely to attribute the robot’s successful performance to the robot itself when they perceive that the robot has intentional agency.

The results of this study can be leveraged by robot designers and developers to make specific trust repair strategies like apologies and denials more effective by encouraging humans to see them as possessing greater degrees of conscious experience. Past research has identified several approaches to encourage humans to view robots as possessing greater degrees of conscious experience. For example, Cuccinielo et al.^93^ compared how a robot’s behavioral style can impact a human’s perception of a robot’s mind. Their findings indicated that when robots adopted a friendly behavioral style humans viewed them as having a greater capacity for conscious experiences^93^. Previous research has shown that manipulating the way one presents and describes robots can encourage humans to view them as capable of having conscious experiences. For instance, Wang and Krumhuber^94^ found that promoting perceptions of a robot’s social value increased the degree to which humans perceived the robot as possessing conscious experience. Researchers have also shown that by manipulating a human’s perceptions of a robot’s capacity for “hunger, fear, and other emotions” designers can encourage humans to view the robot as having more capacity for having a conscious experience^58,]^. Future research can explicitly examine the link between these manipulations and the effectiveness of trust repair strategies, however, questions remain about whether this approach is deceptive. Designing robots to encourage humans to view them as capable of having conscious experiences.

Designing robots to encourage humans to view them as capable of having conscious experiences can be viewed as a type of deception to be avoided rather than encouraged. There are ongoing debates about the problems associated with deceptive robots^95–97^. For example, scholars have argued that robots are genuinely incapable of possessing emotion and that encouraging humans to see them as having this capacity via conscious experience is dishonest and deceptive^96,98–100^. Additionally, there is the possibility that trust repair can be used inappropriately. For example, if a robot is not capable of performing a task better and should not be trusted but offers an effective trust repair humans are likely to trust them when they should not. This can lead to wide-ranging issues in terms of appropriate use and reliance which pose not only psychological risks but also physical risks. This mirrors ongoing debates in the area of explainable AI where tension exists between increasing the communicative efficacy of technology at the expense of deceiving humans about the actual capability of the technology^101–104^. As a result, it is important for designers of robots to consider ethical perspectives related to deception and the inappropriate use of such approaches. That being said, ethical questions around if or when to use such approaches are too nuanced and context-specific to put forth one simple rule of thumb for all situations.

The study findings contribute to the existing literature by identifying a boundary condition where trust repair strategies remain effective despite multiple trust violations. In this study, we investigated the robustness of trust repair strategies relative to mind perception in the context of multiple trust violations. This is particularly relevant because robots are prone to making multiple rather than one error. Prior research has suggested that the effectiveness of trust repair strategies may diminish with repeated trust violations, rendering them ineffective^28^. It would be valuable to determine not only when a trust repair strategy loses effectiveness, but also at what particular trust violation this occurred. As shown in the supplemental material accompanying this paper, our findings contradict previous literature, indicating that when conscious experience is high both apologies and denials remain effective even after multiple trust violations (see Supplementary Fig. S1). This suggests that conscious experience may play a crucial role in establishing conditions for resilient trust repair over multiple trust violations.

This study’s findings also have implications in the field of human–machine communication in two ways. First, the existing literature acknowledges the need for machines to express emotions and sincerity to be effective communicators^105–107^. This is particularly important when machines are attempting to restore or repair relationships with humans after violations^106,108,109^. Scholars have sought to design robots to display emotions during communications with humans to overcome this issue^110^. Nonetheless, other scholars have argued that even with added design features machines are still likely to be perceived as being incapable of feeling or thinking^106,111^. The results of this study, however, bridge the gap between these two opposing views. In particular, individuals’ perceptions of a robot’s conscious experience can help explain when humans see robots as capable of having emotions and as a result are more effective communicators.

Second, these findings can be interpreted as evidence that supports the extension of the expectancy violation theory (EVT) to human–machine communication. EVT is a theory of communication that seeks to explain how individuals respond after experiencing unexpected violations of social norms and expectations^112^. These violations can be either positive or negative violations^113^. Under EVT, an apology from a robot can be viewed as a positive violation because humans do not expect robots to engage in trust repair strategies. The impact of a violation is dependent on the communicator’s reward valence. The communicator’s reward is the degree to which the communicator provides the recipient with what they want or need^112^. These wants or needs could be positive or negative. Positive valence rewards include emotional support, attention, and other indications of engagement. According to Bippus and Young^86^, sincerity can be viewed as a type of communicator’s reward valence in the act of trust repair. The findings of our study provide support for EVT in the context of human–machine communication and by doing so hold theoretical implications outside of only trust repair in HRI but also for theory in the HMC domain.

## Limitations and future work

This paper provides useful insights into the relationships between the effectiveness of trust repairs and human perceptions of robot minds. Regardless, no study is all encompassing and there are several limitations of this work that provide opportunities for future work. First, this study used an online distribution method and an immersive virtual environment. This methodology was adopted as it allowed the study team to overcome numerous limitations related to conducting human subjects research during the COVID-19 pandemic. These included local and federal policies around in-person gatherings, and other legal and health oriented barriers to conducting user studies.

It is possible, however, that a more naturalistic and less controlled environment may have resulted in different trusting behaviors. This is subject to an ongoing debate but, there is increasing support for the use of virtual representations of robots for HRI research^114–120^. Specifically, Deb et al.^114^ found that subjects in virtual environments still interacted similarly to how they did in the real world. Additionally,^117^ directly compared human’s response to physically present robots to human’s responses to virtual representations of robots in multiple forms. Their results indicated no significant differences between physically present and virtually represented robots for eeriness, likability, and purchase intention but did find that human’s perceptions of robot immediacy significantly differed and saw mixed results for human-likeness.

These results are echoed by Gittens et al.^119^ who also compared physically present robots to virtual representations of robots. Their results indicated no significant differences in human’s experience with, perception of, and attitude towards robots between these two interaction modalities. From this they concluded in subsequent work that “there was nothing inherently detrimental to performing HRI user studies online”^120,]^. Nonetheless, we acknowledge this as a potential limitation and future work is needed. Such work should seek to replicate our findings with physical robots in a real-world setting but more generally to also directly compare the use of physically and virtually present robots from a methodological standpoint.

Second, the HRI literature has observed that different tasks, environments, and robots can influence trust between humans and robots^121,122^. As a result, it is possible that with a different task, environment, and robot our results may have been different. The degree of this difference, however, has yet to be fully examined. Additional research is needed to consider how different tasks, environments, and robots might impact this paper’s results. This study also focused primarily on how mind perception impacts trust repair after a specific type of trust violation. Notably, there also exists a range of different types of trust violations. Likewise, future research could be conducted that specifically examines how the type of trust violation (i.e. mistakes) might impact mind perception and the efficacy of different trust repair strategies over time.

Third, our measure of trust propensity was found to be reliable at an $\alpha$ of 0.69. This reliability is acceptable based on^65^ but, the specific cutoff thresholds for reliability often differ between disciplines with an $\alpha > 0.7$ preferred in other domains^123^. Give, however, the recommendations of^65^, the variance among thresholds across disciplines, the conceptual links between trust and trust propensity, and that $\alpha = 0.69$ is within 0.01 of the stricter threshold of $\alpha > 0.7$, the authors of this paper feel that the reliability of $\alpha = 0.69$ justifies the inclusion of trust propensity in our analysis. Regardless, future work may wish to consider an alternative measure of trust propensity or consider modification to this existing measure.

Fourth, it is important to note that different definitions and measures of trust exist in the literature. In this study, we focused our examination on how repairs impact trust as opposed to trustworthiness. To measure trust we used a short 3-item measure originating from the human–human literature but validated for use in HRI and with virtual robots^21^. This measure was selected due to the repeated measures nature of our study’s design. It is worth acknowledging, however, that ongoing debates are present regarding the parity of measures developed for HRI and those developed for human–human interaction^124,125^. Regardless, there is support for adapting human–human trust measures for use in HRI based on the computers as social actors (CASA) paradigm^126–130^. Regardless, using an HRI-specific measure such as the multi-dimensional measure of trust (MDMT)^68^ or similar measures may have further strengthened our findings. Therefore, future work in the HRI domain should consider employing more complex conceptualizations of trust and HRI-specific measurement instruments to build upon the results presented in this study. Such studies could leverage the findings within this paper and contribute to the further development of the field of HRI.

Fifth, we should acknowledge that mind perception can be both conceptualized and measured in many different ways. For example, mind perception has been conceptualized as a uni-dimensional construct^131^ as well as a three-dimensional construct^132^. Furthermore, recent work has also showcased implicit measures of mind perception as alternatives to explicit measures^133^. In this study, we adopted the more commonly implemented 2-dimensional approach to mind perception and examined these dimensions with explicit measures. We did so due to the widely accepted use of this approach across a range of literature in both the human–human and human–robot domain^42–44,51,134–136^. This in turn allows our findings to be directly compared to the existing literature on mind perception.

## Conclusion

This study examines the relationship between mind perception and trust repair in human–robot interaction. Results of this study indicated that overall, individual differences in mind perception are vital considerations when seeking to implement effective apologies and denials between humans and robots. From a broader perspective, this contributes to the growing body of literature on trust repair in HRI by building on existing work examining individual differences and reinforcing not only that individual differences can impact trust repair but that they may do so differently based on what repair strategy is deployed.

## Supplementary Information

Supplementary Information.

## Data Availability

All data generated or analyzed during this study are included in this published article [and its supplementary information files].
