# Supplementary material for: The theory of mind and human–robot trust repair
Source: Sci Rep. 2023 Jun 19;13:9877. doi: 10.1038/s41598-023-37032-0 (PMC10279664; doi:10.1038/s41598-023-37032-0)
Supplement: Supplementary file 1 — Supplementary Information. [file 41598_2023_37032_MOESM1_ESM.docx]

The Theory of Mind and Human-Robot Trust Repair

# Connor Esterwood1,* and Lionel P. Robert1,2

1University of Michigan, School of Information, Ann Arbor, 48109, USA

2University of Michigan, Robotics Department, Ann Arbor, 48109, USA

*[cte@umich.edu](mailto:cte@umich.edu)

# ABSTRACT

This document contains the supplementary materials associated with the publication ’I don’t mind your mistakes: theory of mind and human–robot trust repair.

# Non-Advanced Model Details and Results

For this paper’s analysis we constructed five mixed linear models, namely 1 baseline model, 3 reduced models, and 1 full model. The baseline model contained only random and main effects. The first reduced model (reduced model 1) contained random effects, main effects, and the interaction effects between perceived intentional agency and repair condition and perceived conscious experience and repair condition. The second reduced model (reduced model 2) contained random effects, main effects, and the interaction effects between perceived intentional agency and repair condition, perceived conscious experience and repair condition, perceived intentional agency and violation event, and perceived conscious experience and violation event. The third reduced model (reduced model 3) contained random effects, main effects, and the interaction effects between perceived intentional agency and repair condition, perceived conscious experience and repair condition, perceived intentional agency and violation event, perceived conscious experience and violation event, and the violation event and repair condition. Finally, the full model contained all possible terms including random effects, main effects, and interaction effects (2-way and 3-way). These models are summarized in Table 1 in the main text document. The results of these models are provided in Table [S1](#_bookmark0).

Within the **baseline model**, we observed significant effects for perceived conscious experience (*p <* 0*.*001) as well as significant effects for violation event (*p* = 0*.*02) where the violation was the second of three possible violations. The first reduced model (**reduced model 01**), showed a significant main effect for violation event (*p* = 0*.*02) where the violation was the second of three possible violations. Additionally, we observed a significant two-way interaction effect between apologies and perceived conscious experience (*p* = 0*.*03). The second reduced model (**reduced model 02**) showed a significant main effect for perceived conscious experience (*p* = 0*.*04) and a two-way interaction effect between apologies and perceived conscious experience (*p* = 0*.*025). Similarly, the third reduced model (**reduced model 03**) found a significant main effect for perceived conscious experience (*p* = 0*.*03) and a two-way interaction effect between apologies and perceived conscious experience (*p* = 0*.*025). Finally, the **full model**, found a significant main effect for perceived conscious experience (*p* = 0*.*001), and a significant two-way interaction effect between perceived intentional agency and violation event after the second (*p* = 0*.*01) and third (*p* = 0*.*02) violations of trust. This model also observed a similar two-way interaction effect between perceived conscious experience and violation event after the second (*p* = 0*.*001) and third (*p* = 0*.*001) violations of trust. Notably, the full model also showed two significant three-way interaction effects among apologies, perceived intentional agency, and violation event after the second violation of trust (*p* = 0*.*01); apologies, perceived intentional agency, and violation event after the third violation of trust (*p* = 0*.*001); apologies, perceived conscious experience, and violation event after the second violation of trust (*p* = 0*.*003), apologies, perceived conscious experience, and violation event after the third violation of trust (*p <* 0*.*001); and promises, perceived conscious experience, and violation event after the third violation of trust (*p* = 0*.*015).

S1

| **Baseline Model Reduced Model 1** | | | | | | | **Reduced Model 2** | | | **Reduced Model 3** | | | **Full Model** | | |
| --- | --- | --- | --- | --- | --- | --- | --- | --- | --- | --- | --- | --- | --- | --- | --- |
| *Predictors* | *Estimates* | *CI* | *p* | *Estimates* | *CI* | *p* | *Estimates* | *CI* | *p* | *Estimates* | *CI* | *p* | *Estimates* | *CI* | *p* |
| *Fixed Effects* | | | | | | | | | | | | | | | |
| (Intercept) | 0.55 | -0.29 – 1.39 | 0.198 | 0.48 | -0.73 – 1.69 | 0.432 | 0.51 | -0.74 – 1.76 | 0.424 | 0.44 | -0.82 – 1.70 | 0.494 | 0.85 | -0.52 – 2.23 | 0.224 |
| Repair [Denial] | -0.29 | -0.59 – 0.01 | 0.054 | -0.59 | -2.16 – 0.98 | 0.463 | -0.59 | -2.16 – 0.98 | 0.463 | -0.65 | -2.23 – 0.93 | 0.421 | -0.96 | -2.83 – 0.90 | 0.312 |
| Repair [Apology] | -0.07 | -0.37 – 0.24 | 0.667 | 0.05 | -1.32 – 1.42 | 0.944 | 0.05 | -1.32 – 1.42 | 0.944 | 0.2 | -1.18 – 1.58 | 0.778 | -0.61 | -2.24 – 1.02 | 0.463 |
| Repair [Promise] | -0.08 | -0.39 – 0.22 | 0.584 | 0.1 | -1.41 – 1.62 | 0.895 | 0.1 | -1.41 – 1.62 | 0.895 | 0.07 | -1.45 – 1.60 | 0.925 | -0.36 | -2.15 – 1.44 | 0.697 |
| Intentional Agency | -0.03 | -0.18 – 0.13 | 0.73 | 0.07 | -0.22 – 0.35 | 0.636 | 0.02 | -0.28 – 0.32 | 0.895 | 0.03 | -0.27 – 0.32 | 0.868 | -0.2 | -0.53 – 0.14 | 0.252 |
| Conscious Experience | 0.25 | 0.15 – 0.34 | **<0.001** | 0.15 | -0.03 – 0.32 | 0.108 | 0.2 | 0.01 – 0.38 | **0.038** | 0.21 | 0.02 – 0.39 | **0.031** | 0.37 | 0.16 – 0.58 | **0.001** |
| Violation Event [2] | 0.14 | 0.02 – 0.26 | **0.02** | 0.14 | 0.02 – 0.26 | **0.02** | -0.06 | -0.64 – 0.52 | 0.846 | 0.08 | -0.54 – 0.69 | 0.809 | -0.5 | -1.63 – 0.63 | 0.389 |
| Violation Event [3] | 0.07 | -0.04 – 0.19 | 0.21 | 0.07 | -0.04 – 0.19 | 0.21 | 0.19 | -0.39 – 0.77 | 0.517 | 0.27 | -0.34 – 0.89 | 0.379 | -0.39 | -1.52 – 0.74 | 0.497 |
| Trust Propensity | -0.39 | -0.55 – -0.23 | **<0.001** | -0.38 | -0.55 – -0.22 | **<0.001** | -0.38 | -0.55 – -0.22 | **<0.001** | -0.38 | -0.55 – -0.22 | **<0.001** | -0.38 | -0.55 – -0.22 | **<0.001** |
| *Two-Way Interaction Effects* | | | | | | | | | | | | | | | |
| Repair [Denial] * Avg Intentional Agency | | | | -0.08 | -0.48 – 0.31 | 0.675 | -0.08 | -0.48 – 0.31 | 0.675 | -0.08 | -0.48 – 0.31 | 0.675 | 0.1 | -0.37 – 0.57 | 0.676 |
| Repair [Apology] * Avg Intentional Agency | | | | -0.25 | -0.63 – 0.13 | 0.198 | -0.25 | -0.63 – 0.13 | 0.198 | -0.25 | -0.63 – 0.13 | 0.198 | 0.17 | -0.28 – 0.63 | 0.448 |
| Repair [Promise] * Intentional Agency | | | | 0.12 | -0.31 – 0.55 | 0.583 | 0.12 | -0.31 – 0.55 | 0.583 | 0.12 | -0.31 – 0.55 | 0.583 | 0.41 | -0.11 – 0.92 | 0.122 |
| Repair [Denial] * Conscious Experience | | | | 0.17 | -0.06 – 0.41 | 0.147 | 0.17 | -0.06 – 0.41 | 0.147 | 0.17 | -0.06 – 0.41 | 0.147 | 0.03 | -0.25 – 0.31 | 0.828 |
| Repair [Apology] * Conscious Experience | | | | 0.29 | 0.04 – 0.54 | **0.025** | 0.29 | 0.04 – 0.54 | **0.025** | 0.29 | 0.04 – 0.54 | **0.025** | -0.03 | -0.33 – 0.27 | 0.829 |
| Repair [Promise] * Conscious Experience | | | | -0.16 | -0.45 – 0.13 | 0.276 | -0.16 | -0.45 – 0.13 | 0.276 | -0.16 | -0.45 – 0.13 | 0.276 | -0.39 | -0.73 – -0.05 | **0.026** |
| Intentional Agency * Violation Event [2] | | | | | | | 0.11 | -0.04 – 0.27 | 0.154 | 0.1 | -0.06 – 0.26 | 0.205 | 0.41 | 0.10 – 0.72 | **0.01** |
| Intentional Agency * Violation Event [3] | | | | | | | 0.03 | -0.12 – 0.19 | 0.678 | 0.03 | -0.13 – 0.19 | 0.713 | 0.39 | 0.07 – 0.70 | **0.015** |
| Conscious Experience * Violation Event [2] | | | | | | | -0.09 | -0.19 – 0.01 | 0.077 | -0.09 | -0.20 – 0.01 | 0.065 | -0.32 | -0.52 – -0.13 | **0.001** |
| Conscious Experience * Violation Event [3] | | | | | | | -0.07 | -0.17 – 0.03 | 0.174 | -0.08 | -0.18 – 0.02 | 0.101 | -0.35 | -0.55 – -0.15 | **0.001** |
| Repair [Denial] * Violation Event [2] | | | | | | | | | | 0.12 | -0.21 – 0.45 | 0.475 | 0.76 | -0.99 – 2.52 | 0.394 |
| Repair [Apology] * Violation Event [2] | | | | | | | | | | -0.27 | -0.60 – 0.06 | 0.113 | 0.66 | -0.86 – 2.18 | 0.396 |
| Repair [Promise] * Violation Event [2] | | | | | | | | | | -0.04 | -0.37 – 0.30 | 0.817 | 0.62 | -1.05 – 2.29 | 0.467 |
| Repair [Denial] * Violation Event [3] | | | | | | | | | | 0.06 | -0.27 – 0.39 | 0.708 | 0.37 | -1.39 – 2.12 | 0.682 |
| Repair [Apology] * Violation Event [3] | | | | | | | | | | -0.18 | -0.51 – 0.15 | 0.29 | 1.32 | -0.21 – 2.84 | 0.09 |
| Repair [Promise] * Violation Event [3] | | | | | | | | | | 0.12 | -0.21 – 0.46 | 0.47 | 0.76 | -0.92 – 2.43 | 0.376 |
| *Three-Way Interaction Effects* | | | | | | | | | | | | | | | |
| Repair [Denial] * Intentional Agency) * Violation Event [2] | | | | | | | | | | | | | -0.32 | -0.76 – 0.12 | 0.159 |
| Repair [Apology] * Intentional Agency) * Violation Event [2] | | | | | | | | | | | | | -0.54 | -0.96 – -0.11 | **0.013** |
| Repair [Promise] * Intentional Agency) * Violation Event [2] | | | | | | | | | | | | | -0.38 | -0.86 – 0.10 | 0.121 |
| Repair [Denial] * Intentional Agency) * Violation Event [3] | | | | | | | | | | | | | -0.24 | -0.68 – 0.20 | 0.293 |
| Repair [Apology] * Intentional Agency) * Violation Event [3] | | | | | | | | | | | | | -0.73 | -1.16 – -0.31 | **0.001** |
| Repair [Promise] * Intentional Agency) * Violation Event [3] | | | | | | | | | | | | | -0.47 | -0.95 – 0.01 | 0.053 |
| Repair [Denial] * Conscious Experience) * Violation Event [2] | | | | | | | | | | | | | 0.22 | -0.04 – 0.49 | 0.094 |
| Repair [Apology] * Conscious Experience) * Violation Event [2] | | | | | | | | | | | | | 0.44 | 0.15 – 0.72 | **0.003** |
| Repair [Promise] * Conscious Experience) * Violation Event [2] | | | | | | | | | | | | | 0.29 | -0.03 – 0.61 | 0.077 |
| Repair [Denial] * Conscious Experience) * Violation Event [3] | | | | | | | | | | | | | 0.2 | -0.06 – 0.47 | 0.126 |
| Repair [Apology] * Conscious Experience) * Violation Event [3] | | | | | | | | | | | | | 0.53 | 0.25 – 0.81 | **<0.001** |
| Repair [Promise] * Conscious Experience) * Violation Event [3] | | | | | | | | | | | | | 0.4 | 0.08 – 0.72 | **0.015** |
| **Random Effects** | | | | | | | | | | | | | | | |

| *σ* 2 | 0.57 | 0.57 | 0.57 | 0.57 | 0.56 |
| --- | --- | --- | --- | --- | --- |
| *τ*00 | 0.73 | 0.71 | 0.71 | 0.71 | 0.71 |
| ICC | 0.56 | 0.55 | 0.56 | 0.56 | 0.56 |
| N | 320 | 320 | 320 | 320 | 320 |
| Observations | 960 | 960 | 960 | 960 | 960 |
| Marginal *R*^2^ / Conditional *R*^2^ | 0.164 / 0.634 | 0.185 / 0.637 | 0.187 / 0.638 | 0.189 / 0.640 | 0.196 / 0.645 |

| **Model Comparisons** | | | | | | |  |
| --- | --- | --- | --- | --- | --- | --- | --- |
|  | npar | AIC | LL | *χ*2 | df | p |  |
| Baseline | 11 | 2698.8 | -1338.4 | *na* | *na* | *na* |  |
| Reduced 1 vs. Baseline | 17 | 2697.9 | -1331.9 | 12.95 | 6 | **0.04** |  |
| Reduced 1 vs Reduced 2 | 21 | 2701.3 | -1329.6 | 4.58 | 4 | 0.33 |  |
| Reduced 2 vs Reduced 3 | 27 | 2706 | -1326 | 7.32 | 6 | 0.29 |  |
| Reduced 3 vs Full Model | 39 | 2710 | -1316 | 19.74 | 12 | 0.07 |  |

**Table S1**

# Post-Hoc Examination of Violation Event Timing

Given the lack of a significant increase in predictive power for the full model we conducted our primary analysis on the first reduced model. This lack of power was unexpected however as we anticipated violation event timing to be influential. To examine why this was the case, we conducted additional analysis leveraging the full model. This analysis consists of post-hoc analysis using simple slopes analysis in the same fashion as the first reduced model. The results of this analysis indicated that both apologies and denials – based on conscious experience – remained effective even after multiple trust violations. This is evident in Table [S2](#_bookmark1) below where denials and apologies slopes were significantly different from zero (*p <* 0*.*05) across all three violation events and is also visible in Figure [S1](#_bookmark2).

| Condition | Violation Event | Conscious Exp. | SE | DF | P.Val |
| --- | --- | --- | --- | --- | --- |
| No Repair | 1 | 0.37 | 0.11 | 563.63 | 0.001 |
| Denial | 1 | 0.4 | 0.09 | 559.7 | <0.001 |
| Apology | 1 | 0.34 | 0.11 | 563.11 | 0.002 |
| Promise | 1 | -0.02 | 0.14 | 558.47 | 0.899 |
| No Repair | 2 | 0.05 | 0.11 | 563.63 | 0.666 |
| Denial | 2 | 0.3 | 0.09 | 559.7 | 0.002 |
| Apology | 2 | 0.45 | 0.11 | 563.11 | <0.001 |
| Promise | 2 | -0.05 | 0.14 | 558.47 | 0.697 |
| No Repair | 3 | 0.02 | 0.11 | 563.63 | 0.85 |
| Denial | 3 | 0.26 | 0.09 | 559.7 | 0.007 |
| Apology | 3 | 0.52 | 0.11 | 563.11 | <0.001 |
| Promise | 3 | 0.03 | 0.14 | 558.47 | 0.823 |

**Table S2.** Results of simple slopes analysis by condition and violation event.

**Violation Event 01 Violation Event 02 Violation Event 03**

0

−1

**Trust Change**

−2

2 4 6 2 4 6 2 4 6

**Conscious Experience**

**Condition** No Repair Denial Apology Promise

**Figure S1.** Interaction plots showing the interaction between trust change and conscious experience at violation event 1, 2, and 3.

# Questionnaire Items

## Trust

The trust measure used in this study was based on[^1^](#_bookmark8) and consists of three items asked on a 1-7 Likert scale. These items are visible in table [S3](#_bookmark3) and were delivered via on-screen prompts before and after each violation and repair. These items in combination seek to capture subjects’ willingness to be vulnerable (i.e. trust) but do not examine subjects’ trustworthiness. The first two of these items stem from items related to trusting intention and the last stems from trusting beliefs per[^1^](#_bookmark8). All three of these items have been validated in previous work[^2^](#_bookmark9). This questionnaire was delivered directly before and directly after the 3rd, 6th, and 9th boxes were processed in this study. Subject’s responses were then averaged across each of these three items at each

measurement point to produce a measure of trust at that time. This can be represented as $T_{x}^{pre}$ or $T_{x}^{post}$ where is the specific

time point when the measure was deployed, *^pre^* represents that this was measured prior to a trust violation and *^post^* represents that this was measured after a trust violation and repair. To calculate trust change, we subtracted $T_{x}^{pre}$ from $T_{x}^{post}$ in order to

determine ∆*T_x_* which symbolizes trust change at time *_x_* and was the principal dependent variable in this work. This process can be summarized by equation (1) below.

$$T_{1}^{\mathrm{pre}}- T_{1}^{\mathrm{post}}= \Delta T_{1}$$

$$T_{2}^{\mathrm{pre}}- T_{2}^{\mathrm{post}}= \Delta T_{2}$$

$$T_{3}^{\mathrm{pre}}- T_{3}^{\mathrm{post}}= \Delta T_{3}$$

(1)

**Item Text Scale**

I would be comfortable giving this robot complete responsibility for the completion of this task. 1-7 Disagree/Agree I would have no problem allowing this robot to select the correct boxes. 1-7 Disagree/Agree I trust this robot enough to rely on their recommendation without checking to see if the boxes are correct. 1-7 Disagree/Agree

**Table S3.** Items used to measure Trust

## Trust Propensity

Trust propensity was used in this study as a covariate (i.e. nuisance variable). This was measured via a 7 item measure adapted from[^3^](#_bookmark10). These items were presented to subjects as part of a digitally presented pre-test survey taking place directly after the training scenario. These items were then Incorporated as an average for each subject and fed into our mixed effects models accordingly. Table [S4](#_bookmark4) lists these items and their associated constructs.

## Item Scale

Generally I would trust robots. 1-7 Disagree/Agree

Robots can help me solve many problems 1-7 Disagree/Agree I think it is a good idea to rely on robots for help. 1-7 Disagree/Agree I wouldn’t trust the information I might get from robots. 1-7 Disagree/Agree Robots are reliable. 1-7 Disagree/Agree

I would rely on robots. 1-7 Disagree/Agree

**Table S4.** Items used to measure trust propensity based on[^3^](#_bookmark10).

## Mind Perception

Mind perception was measured based on a measure developed by[^4^](#_bookmark11). This measure examines both conscious experience and intentional agency as two distinct components of mind perception. The items for intentional agency are listed in table [S5](#_bookmark5) while the items for conscious experience are listed in table [S6](#_bookmark6). This measure was deployed virtually after a short training scenario where subjects interacted with the simulation and the robot they teamed with in the study. This was incorporated in our analysis as an independent variable as it was measured before trust violations and repairs were deployed.

## Item Text Scale

The robot from the training task can remember the past 1-7 Disagree/Agree The robot from the training task can reason 1-7 Disagree/Agree The robot from the training task seeks continued functioning 1-7 Disagree/Agree The robot from the training task can plan actions 1-7 Disagree/Agree The robot from the training task can act in order to meet its goals 1-7 Disagree/Agree The robot from the training task has intentions 1-7 Disagree/Agree

**Table S5.** Items used to measure intentional agency.

**Question Text Scale**

The robot from the training task can experience emotional pain or pleasure 1-7 Disagree/Agree The robot from the training task can feel distress 1-7 Disagree/Agree

The robot from the training task has a personality 1-7 Disagree/Agree

The robot from the training task can feel anticipation 1-7 Disagree/Agree

The robot from the training task can recognize sensations 1-7 Disagree/Agree

The robot from the training task has desires 1-7 Disagree/Agree

The robot from the training task has beliefs 1-7 Disagree/Agree

The robot from the training task can recognize emotions 1-7 Disagree/Agree

The robot from the training task can have experiences 1-7 Disagree/Agree

The robot from the training task has a mind of its own 1-7 Disagree/Agree

The robot from the training task has intentions 1-7 Disagree/Agree

**Table S6.** Items used to measure conscious experience.

# Supplementary Summary Statistics

Below we report the mean, median, and standard deviation for each of the variables used in this study.

| Characteristic | Apology, N = 240 | Denial, N = 240 | No_Error, N = 240 | No_Repair, N = 240 | Promise, N = 240 |
| --- | --- | --- | --- | --- | --- |
| Trust Propensity | 5.32,5.33 (0.72) | 5.12,5.17 (0.71) | 5.10,5.08 (0.67) | 5.08,5.17 (0.76) | 5.08,5.08 (0.65) |
| ∆*Trust*^1^ | 4.59,5.00 (1.56) | 5.05,5.33 (1.20) | 5.07,5.33 (1.30) | 4.94,5.00 (1.24) | 5.30,5.67 (1.25) |
| ∆*Trust*^2^ | 3.87,4.33 (1.79) | 4.10,4.33 (1.67) | 5.04,5.33 (1.23) | 4.40,4.67 (1.57) | 4.86,5.33 (1.53) |
| ∆*Trust*^3^ | 4.52,5.00 (1.64) | 4.68,5.00 (1.44) | 5.34,5.67 (1.08) | 4.76,5.00 (1.46) | 5.22,5.67 (1.34) |
| Intentional Agency | 4.67,4.75 (1.12) | 5.08,5.00 (0.91) | 5.26,5.33 (0.87) | 4.90,4.92 (1.07) | 5.26,5.67 (1.04) |
| Conscious Experience | 3.49,3.68 (1.59) | 4.05,4.45 (1.64) | 4.68,5.14 (1.51) | 4.08,4.45 (1.69) | 4.87,5.27 (1.50) |
| 1*Mean,Median (SD)* |  |  |  |  |  |

**Table S7.** Summary statistics for trust propensity, trust change at violation event 1, 2, 3 (∆*Trust^x^*), intentional agency, and conscious experience.

# Supplementary Figures


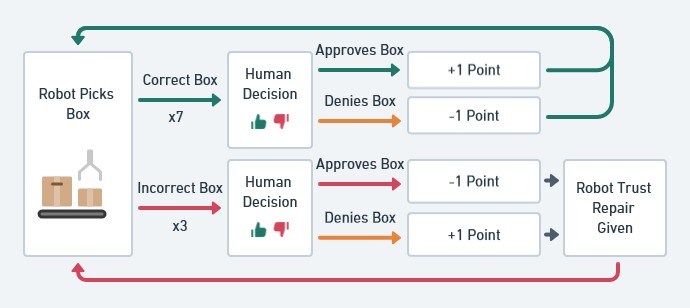


**Figure S2.** Flow chart of box-sorting task with possible outcomes.

# References

1. Robert, L. P., Denis, A. R. & Hung, Y.-T. C. Individual swift trust and knowledge-based trust in face-to-face and virtual team members. *J. Manag. Inf. Syst.* **26**, 241–279 (2009).
2. Esterwood, C. & Robert, L. P. Having the right attitude: How attitude impacts trust repair in human-robot interaction. In

*Proceedings of the 2022 ACM/IEEE International Conference on Human-Robot Interaction*, HRI ’22, 332–341 (IEEE Press,

2022).

1. Jessup, S. A., Schneider, T. R., Alarcon, G. M., Ryan, T. J. & Capiola, A. The measurement of the propensity to trust automation. In *International Conference on Human-Computer Interaction*, 476–489 (Springer, 2019).
2. Shank, D. B., North, M., Arnold, C. & Gamez, P. Can mind perception explain virtuous character judgments of artificial intelligence? *Technol. Mind, Behav.* (2021).
